# Role of Recognition MicroRNAs in *Hemaphysalis longicornis* and *Theileria orientalis* Interactions

**DOI:** 10.3390/pathogens13040288

**Published:** 2024-03-28

**Authors:** Jin Luo, Yangchun Tan, Shuaiyang Zhao, Qiaoyun Ren, Guiquan Guan, Jianxun Luo, Hong Yin, Guangyuan Liu

**Affiliations:** 1 State Key Laboratory for Animal Disease Control and Prevention, Key Laboratory of Veterinary Parasitology of Gansu Province, Lanzhou Veterinary Research Institute, Chinese Academy of Agricultural Science, Xujiaping 1, Lanzhou 730046, China; lxt9093@163.com (Y.T.); zhaoshuaiyang@caas.cn (S.Z.); renqiaoyun@caas.cn (Q.R.); guanguiquang@caas.cn (G.G.); luojianxun@caas.cn (J.L.); 2 MOE Joint International Research Laboratory of Animal Health and Food Safety, College of Veterinary Medicine, Nanjing Agricultural University, Nanjing 210095, China; 3 Jiangsu Co-Innovation Center for the Prevention and Control of Important Animal Infectious Disease and Zoonosis, Yangzhou University, Yangzhou 225009, China

**Keywords:** ticks, longipain, miR-5309, *Theileria orientalis*, *Hemaphysalis longicornis*, interactions

## Abstract

Ticks are an important type of pathogen transmission vector, and pathogens not only cause serious harm to livestock but can also infect humans. Because of the roles that ticks play in disease transmission, reducing tick pathogen infectivity has become increasingly important and requires the identification and characterization of these pathogens and their interaction mechanisms. In this study, we determined the miRNA expression profile of *Hemaphysalis longicornis* infected with *Theileria orientalis*, predicted the target genes of miRNAs involved in this infection process, and investigated the role of miRNA target recognition during host–pathogen interactions. The results showed that longipain is a target gene of miR-5309, which was differentially expressed at different developmental stages and in various tissues in the control group. However, the miR-5309 level was reduced in the infection group. Analysis of the interaction between miRNA and the target gene showed that miR-5309 negatively regulated the expression of the longipain protein during the infection of *H. longicornis* with *T. orientalis*. To verify this inference, we compared longipain with the blocking agent orientalis. In this study, the expression of longipain was upregulated by the inhibition of miR-5309 in ticks, and the ability of the antibody produced by the tick-derived protein to attenuate *T. orientalis* infection was verified through animal immunity and antigen–antibody binding tests. The results showed that expression of the longipain + GST fusion protein caused the cattle to produce antibodies that could be successfully captured by ticks, and cellular immunity was subsequently activated in the ticks, resulting in a subtractive effect on *T. orientalis* infection. This research provides ideas for the control of ticks and tickborne diseases and a research basis for studying the mechanism underlying the interaction between ticks and pathogens.

## 1. Introduction

*Theileria orientalis* (*T. orientalis*) is widely distributed and causes theileriosis, a condition in which the pathogen invades and destroys red blood cells of the host after entering via a tick bite [1,2], and *T. orientalis* is mainly transmitted by the *Hemaphysalis longicornis* (*H. longicornis*) vector [3]. *T. orientalis* is transmitted throughout a tick’s lifetime. *H. longicornis*, which enters the midgut of ticks and invades intestinal epithelial cells, feeds on the blood of infected *T. orientalis*. When *H. longicornis* molts and develops to the next stage, *T. orientalis* escapes from the intestine, enters the hemocoel, and ultimately invades the salivary gland. Subsequently, when ticks suck blood, *T. orientalis* can be transmitted to the host [4]. The life cycle of *T. orientalis* is similar to that of other Theileria species, and the fission body does not undergo transformation or lethal lymphoid proliferation [5].

Ticks are blood-sucking arthropods that carry a wide range of pathogens, including viruses, bacteria, protozoa, fungi, and nematodes [6]. These pathogens are transmitted to hosts during the blood-sucking process. Tick-borne diseases, such as forest encephalitis virus, anaplasmosis, and babesiosis, have caused great economic losses worldwide. The salivary gland of *H. longicornis* contains various bioactive molecules that can regulate the immune and inflammatory responses of the host [7], including defensins, microplusin/hebraein, and lysozymes, which promote the acquisition and transmission of pathogens [8,9].

MicroRNAs (miRNAs) are a class of endogenous noncoding small RNAs of approximately 18–22 nt. A microRNA can exert its functions, such as cell development, immune response, and disease occurrence, by binding to the 3′-UTR or 5′-UTR of target genes (mRNAs) at the posttranscriptional level [10,11,12,13,14]. The main reason is that mRNAs play an important role in the pathogenesis of *T. orientalis* [15,16]. Over the years, an increasing body of evidence has shown that miRNAs weaken pathogen replication by regulating the expression of host resistance-related genes or pathogen genes and thus play key roles in host–pathogen interactions [17,18]. miRNAs also control bacterial, parasitic, and viral infections by regulating proteins associated with innate and acquired immune pathways, and pathogens can promote infection and proliferation by modifying host miRNA profiles [19,20,21,22]. Based on their functions, miRNAs have been widely used in the detection, prevention, and control of diseases and in other medical fields. Some studies have shown that miRNAs can regulate the immune system during bacterial infection. This is a reason for many pathogens to modify and evade host immune responses via miRNA regulatory mechanisms [23,24,25]. Multiomics methods provide the possibility for the discovery of numerous immune genes and corresponding regulatory factors. Researchers have shown that when Oncorhynchus mykiss is infected with a hematopoietic crossing virus, transcriptome and miRNA expression profile analysis of the host during virus infection indicates that viral invasion can promote differences in the expression of 3355 transcripts and more than 80 miRNAs in the host. The most significant differences among these factors are found for TLR7/8, which are regulated by miR-143; STAT1, which is targeted by miR-203; and DHX58, which is targeted by miR-181 and inhibits virus invasion [26]. Research has shown that miR-34c-3p plays a regulatory role in cAMP-independent regulation of host cell protein kinase A (PKA) activity in *Theileria annulata*-infected bovine red blood cells. The discovery of this mechanism has important reference value for the prevention and control of diseases caused by *Theileria annulata* [27]. Although research on miRNA regulation of target gene function is still very limited, existing studies have confirmed that miRNA molecules can play an important regulatory role in pathogenic infections of the host [28].

At present, research on the function of the longipain gene is very limited; research indicates that the gene has a structure similar to that of cysteine proteins, and it may have similar functions. Few studies have shown that the longipain gene can be expressed in the midgut epithelial cells of ticks and that its expression is upregulated by feeding on the host’s blood. Enzyme activity measurements indicate that longipain can still undergo hydrolysis within a wide range of pH values. A blood parasite killing test revealed that longipain can effectively kill *Babesia* parasites in ticks, and its killing effect is generated by specific adhesion to the parasite membrane. RNAi experiments confirmed that longipain is involved in blood digestion [29].

In the present study, we aimed to investigate the expression patterns of miRNAs in *H. longicornis* infected or not infected with *T. orientalis*. This study provides an opportunity for elucidating the miRNA-mediated regulation of *T. orientalis* invasion in ticks and other hosts.

## 2. Materials and Methods

### 2.1. Ethics Approval

In this study, cattle and rabbits were handled according to the Animal Ethics Procedures and Guidelines of the People’s Republic of China. This study was approved by the Animal Administration and Ethics Committee of Lanzhou Veterinary Research Institute, Chinese Academy of Agricultural Sciences (approval No. LVRIAEC 2024-006).

### 2.2. Tick Collection and RNA Extraction

In the laboratory, different developmental stages of *H. longicornis* were cultured on rabbits. The eggs produced by engorged adult ticks were kept in sterile tubes and incubated at room temperature and 85% relative humidity. Approximately 3.0 g of larvae was divided into two parts. One was used for RNA extraction, and the other was used for further cultivation until the nymphs hatched. RNA was extracted to maintain the purity of the ticks. The necessary samples were rinsed twice with PBS, followed by cleaning with 0.133 M NaCl, 1.11% sodium dodecyl sulfate (SDS), and 0.0088 M ethylenediaminetetraacetic acid (EDTA) to remove external bacteria from the ticks [30].

The expression of genes associated with different developmental stages of *H. longicornis* was analyzed, and the ticks were ground and crushed in liquid nitrogen for total RNA extraction. Small RNAs (sRNAs) were enriched using a miScript miRNA isolation kit (QIAGEN, Shanghai, China). The integrity of the total RNA was assessed using an Agilent 2100 Bioanalyzer system (Agilent Technologies, Santa Clara, CA, USA). Total RNA was stored at −80 °C until use [29].

### 2.3. Construction of the Tick Infection Model

In this study, clean cattle were obtained from Piroplasmida-free areas and inspected for Piroplasmida in the laboratory at the Lanzhou Veterinary Research Institute. The spleen was removed from the cattle by surgery, and the cattle were randomly divided into two groups for the infection experiments. One group of cattle was inoculated with *T. orientalis* obtained from the Insect Species Collection Center of Lanzhou Veterinary Research Institute, Chinese Academy of Agricultural Science, and the other group was inoculated with 30 nonengorged and clean adult ticks. The *T. orientalis* infection rate reached approximately 10%, as determined by blood smear microscopy. In the experiment, nonengorged *H. longicornis* (larvae, nymphs, and adults) were released into a cloth bag, which was pasted to the back of the cattle, and the other group of cattle was exposed to clean ticks in a similar manner. Approximately 6–8 days later, the engorged ticks were collected from the bags, and these ticks were allowed to develop to the next physiological stage; the miRNAs from these ticks were sequenced, and the miRNA molecules associated with the infection process were examined.

### 2.4. High-Throughput Sequencing of Small RNAs

The sRNA library was constructed according to MGIEasy instructions (MGI Co., Shenzhen, China) [31]. The RNA fragments with a length of 18–50 nt were isolated by size on a 15% TBE (Tris–borate–EDTA–urea) polyacrylamide gel. The 5′ or 3′ adapter was ligated to the sRNA fragments with T4 RNA ligase (Promega, Madison, WI, USA), increasing the length to 41–76 nt. The extended fragment was used for the synthesis of a single strand of cDNA using M-MuLV (Invitrogen, Waltham, MA, USA). The cDNAs were amplified for sRNAs through 35 PCR cycles using stem–loop primers with sRNAs. The PCR products were purified and then sequenced using a HiSeq2000 sequencer.

### 2.5. Small RNA Analysis

Here, the software developed by BGI was used for HiSeq raw data analysis, and adapter sequences, low-quality reads, and duplicate reads were eliminated from these raw data to obtain the final clean reads. These clean reads were compared with tick miRNA precursors or mature miRNAs from other species to obtain miRNA quantity (expression level) or base characteristics of miRNA sequences. The presence of rRNA, scRNA, snoRNA, snRNA, and tRNA was annotated using the GenBank and Rfam databases. To ensure the uniqueness of the sRNA annotations, the following priority order was used: GenBank > Rfam > known miRNA > repeat sequence > exit > intron. The qualified sample standard was the rRNA ratio, which should be below 60% and 40% for plants and animals, respectively.

### 2.6. miRNA Targets and Gene Ontology Analysis

Currently, there is no 3′-UTR database available. Therefore, to predict the target genes of miRNAs, this study used the NCBI database (https://www.ncbi.nlm.nih.gov, accessed on 5 March 2023). The EST database was used to predict the target genes of miRNAs by Allen et al. (2005) [32] and Schwab (2005) [33]. The prediction of miRNA target genes followed the following rules: the mismatch between miRNA and the target should not exceed four bases, the number of mismatched bases between miRNA and target gene should be less than two, there should be no adjacent mismatch between miRNA and target gene at positions 2–12, and the minimum free energy for miRNA matching with the target gene should be ≥75%. In this study, the functions of the candidate target genes were enriched using the Gene Ontology (GO) database (http://www.geneontology.org/, accessed on 5 March 2023) [34].

### 2.7. Real-Time Quantitative PCR

The stem–loop primer (5′-GTC GTA TCC AGT GCA GGG TCC GAG GTA TTC GCA CTG GAT ACG ACT TCG GGG T-3′) was used for miRNA sequence extension [35], facilitating primer design and PCR amplification. According to the universal sequence of the stem–loop primers, the reverse primer for this miRNA was obtained from QIAGEN Co., Ltd., China, and the forward primer for miR5309 (5′-ACA CTC CAG CTG GCA ATG CCC ATG GAA-3′) was designed by Primer Premier 5.0. As an internal reference gene, β-*actin* was used in the qPCR system with the following primers: sense primer, 5′-CGT TCC TGG GTA TGG AAT CG-3′, and antisense primer, 5′-TCC ACG TCG CAC TTC ATG AT-3′ (Table 1). These primers were synthesized by Shenggong Co., Ltd., Shanghai, China. RT–qPCR was performed using an Mx3000pTM SYBR Green (QIAGEN Biotechnology Co., Ltd., China). The reaction mixture was incubated at 37 °C for 60 min and 95 °C for 5 min. The cDNA products were stored at −20 °C.

qPCR was performed with a miScript SYBR Green PCR kit. The miRNA levels in each sample were measured using the 2^−∆∆CT^ method. One-way ANOVA was used to analyze the significance of differences in miRNA expression in various samples. 

### 2.8. Analysis of the Dual-Luciferase Reporter System

The binding site sequences between the miRNAs and target genes were inserted into the pmirGLO reporter vector (Promega, Madison, WI, USA). Next, 293T cells were cotransfected with 150 ng pmirGLO and 50 nM miR-5309 mimetic (RiboBio, Guangzhou, China) using Lipofectamine 3000. Untreated cells were used as blank controls. Cells harboring the pmirGLO-Hsp vector alone were used as negative controls. The cells were cultured at 37 °C in a 5% CO_2_ incubator. After 48 h, the interaction effect between the two was detected, and all samples to be tested were tested with three replicates.

### 2.9. Statistical Analysis

All experimental data were analyzed using Student’s *t* test with GraphPad Prism 5.0. Probability values less than 0.05 were considered to indicate statistical significance, and the results are shown as the means ± SEMs.

## 3. Results

### 3.1. Small RNA Library Construction and Solexa Sequencing

**Figure 1:**
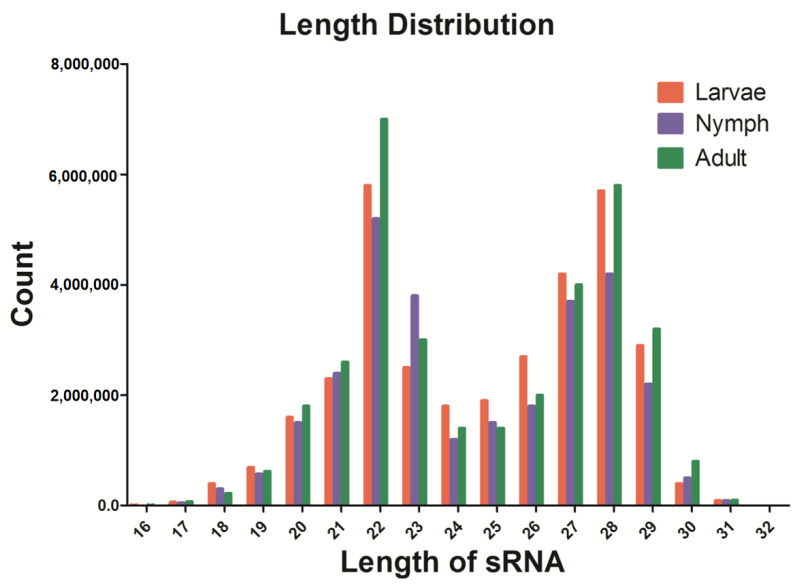
Length and expression abundance statistics of clean sRNAs. The *X*-axis represents the length of sRNAs, and the *Y*-axis represents the copy number of sRNAs.

In this study, sRNA libraries were constructed for the identification of miRNAs in young and adult ticks infected with *Taylonychus orientalis*, and the Illumina HiSeq2000 platform was used for sequencing. The raw sequencing data were used to remove contaminants, splice sequences, and repeat sequences to obtain the final clean reads. SOAP software was used to map clean sRNAs to animal genomes and known miRNAs in the miRBase21 database (http://www.mirbase.org/, accessed on 5 March 2023). The program and parameters were as follows: SOAP-v 0-r 2-M 0-a clean.fa-D ref_genome. fa.index-o match_genome SOAP. The results showed a total of 30,713,634, 19,184,633, and 29,186,165 clean reads of 18–30 nt in the larva, nymph, and adult samples, respectively. The length distribution revealed that most reads were 20–29 nt in length; moreover, the highest percentage of reads (14.13%) had a length of 22 nt, and 20.08% of the sequences were 28 nt long (Figure 1).

### 3.2. Differential Expression Analysis

In this study, known miRNAs obtained from *H. longicornis* ticks infected with *T. orientalis* were analyzed based on miRBase21 data. The results revealed a total of 24,136 known differentially expressed miRNAs in the larval stage (*p* value < 0.01), which included 11,432 downregulated miRNAs and 12,704 upregulated miRNAs. In the nymphal stage, 17,320 miRNAs were downregulated, and 5399 miRNAs were upregulated. In the adult stage, 14,211 miRNAs were downregulated, and 17,770 miRNAs were upregulated. Analysis of miRNA expression abundance at different developmental stages revealed that the copy numbers of miR-1, miR-5309, let-7, etc., were greater than 100,000, and these miRNAs accounted for 17.78% (260,875/1,467,411) of the total reads and exhibited similar expression patterns (upregulated or downregulated) at different developmental stages of tick infection (Table 2), suggesting that they perform important functions during this period. Here, we found that miR-5309 may play an important role in tick infection.

### 3.3. GO Enrichment Analysis of the miR-5309 Target Genes

**Figure 2:**
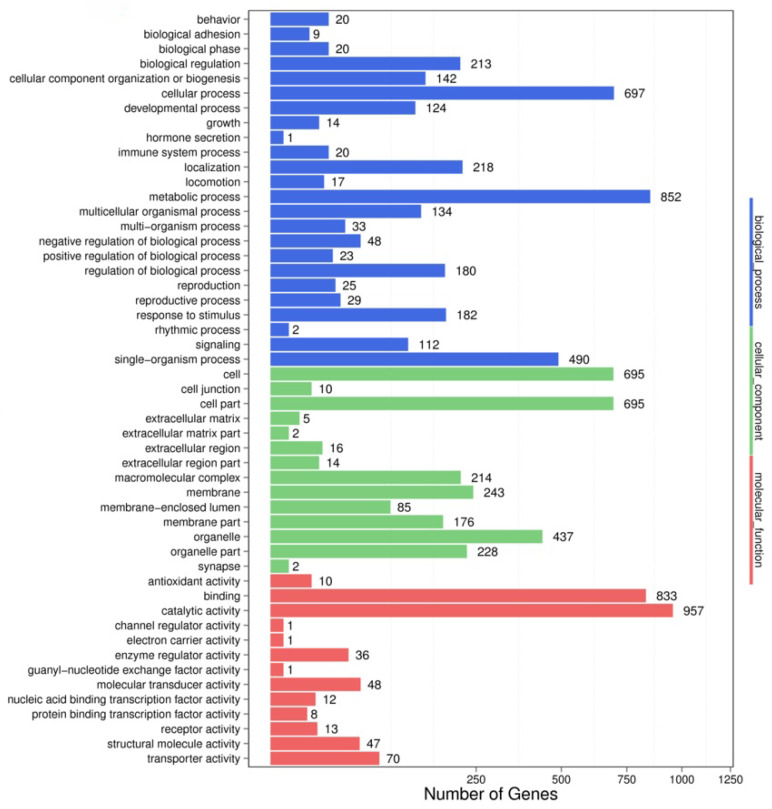
GO enrichment analysis of longipain, a target gene of miR-5309. The functional annotations obtained from the GO analysis were divided into three main categories: molecular function, cellular component, and biological process.

To obtain more accurate results, we used RNAhybrid and miRanda to predict the target genes. In the comparison of the control group with the experimental group at different developmental stages, a total of 6794 target genes of 53 miRNAs were predicted and successfully assigned to 7582 GO terms. The target genes were significantly enriched in GO terms related to the regulation of biological processes, namely biological regulation, humoral immune response, immune response, negative regulation of biological processes, immune system processes, defense responses, gene silencing, and negative regulation of macromolecular metabolic processes (Figure 2). The target genes for miR-5309 were enriched in the negative regulation of biological processes, such as the immune response and defense response.

### 3.4. Analysis of the Expression of miR-5309 and Its Target Longipain in Infected Ticks

**Figure 3:**
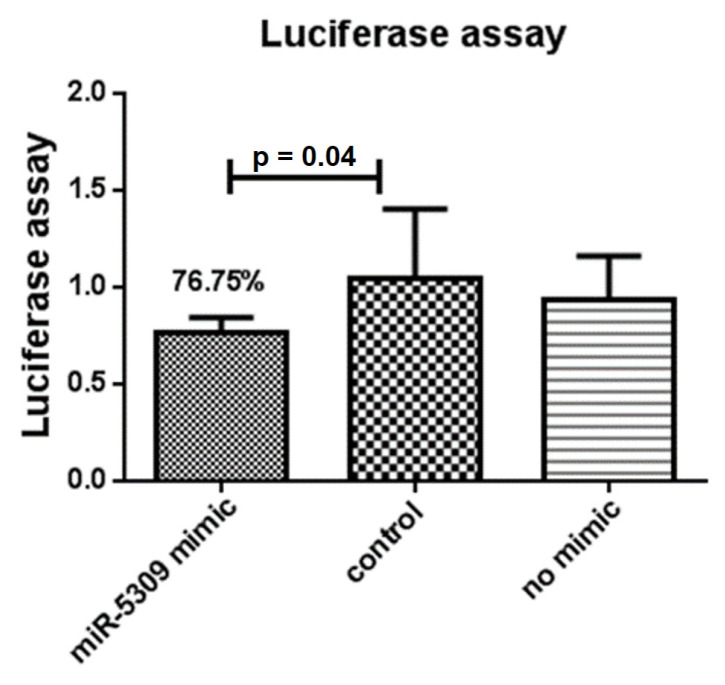
A dual-luciferase reporter system was used to verify miR-5309 and its target gene longipain in vitro. Compared with that of the control group, the fluorescence of the experimental group supplemented with the mimic and recombinant plasmids decreased by 23.25%. A *p* value = 0.04 indicated a significant difference. Subsequently, in vitro experiments verified that the miR-5309 and longipain genes are mutual targets.

The interaction between the target gene longipain and miR-5309 was predicted, and its binding sites were analyzed (Table 3) using RNAhybrid (https://bibiserv.cebitec.uni-bielefeld.de/rnahybrid, accessed on 5 March 2023). Based on these results, a binding site was cloned and inserted into the pmirGLO vector, which was then cotransfected with the miR-5309 mimetic into 293T cells. Compared with the negative control and no simulated control, 76.50% of the luciferase activity was generated at site 1122 (Figure 3). Analysis of another locus showed no significant difference compared to the control. Therefore, longipain has been identified as a potential target for miR-5309 in vitro.

**Figure 4:**
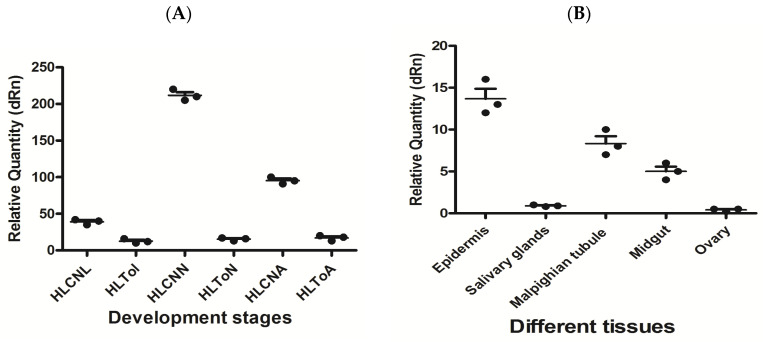
The expression level of miR-5309 before and after infection of ticks at different developmental stages and in various tissues was determined. (**A**) miR-5309 in infected *Hemaphysalis longicornis* at different developmental stages. (**B**) The expression level of miR-5309 in different *H. longicornis* tissues was determined. Note: “HL” is *Hemaphysalis longicornis*; “To” refers to *Theileria orientalis*; “CN” is the control; “L” is larvae; “N” is nymphs; “A” refers to adults.

**Figure 5:**
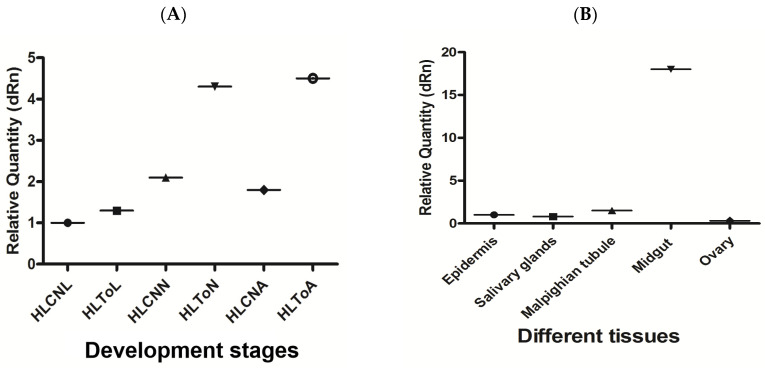
The expression levels of longipain at different developmental stages and in various tissues of ticks before and after infection were determined. (**A**) Longipain expression in infected *Hemaphysalis longicornis* at different developmental stages. (**B**) The expression level of longipain in different tissues of *H. longicornis* was determined. Note: “HL” is *Hemaphysalis longicornis*; “To” refers to *Theileria orientalis*; “CN” is the control; “L” is larvae; “N” refers to nymphs; “A” is the abbreviation for adults.

In this study, the expression levels of miR-5309 and its target genes were detected by RT–qPCR in *H. longicornis* before and after infection with *T. orientalis*. The results showed that the expression of miR-5309 was highest in nymphs before infection, and in these nymphs, the miR-5309 expression level was significantly lower than that in infected ticks (copy number = 200 before infection; copy number = 8 after infection) (Figure 4A). The expression of miR-5309 in different tissues was assessed and was clearly high in the epidermis and Malpighian tubules. However, miR-5039 was expressed at lower levels in the salivary gland and midgut than in the epidermis and Malpighian tubules, which are important immune organs in ticks (Figure 4B). The expression level of the target gene longipain was significantly increased in ticks infected with *T*. *orientalis*. The expression of longipain in the midgut was significantly greater than that in other tissues (Figure 5A,B).

### 3.5. Effects of miRNA-Blocking Inhibition on Ticks

**Figure 6:**
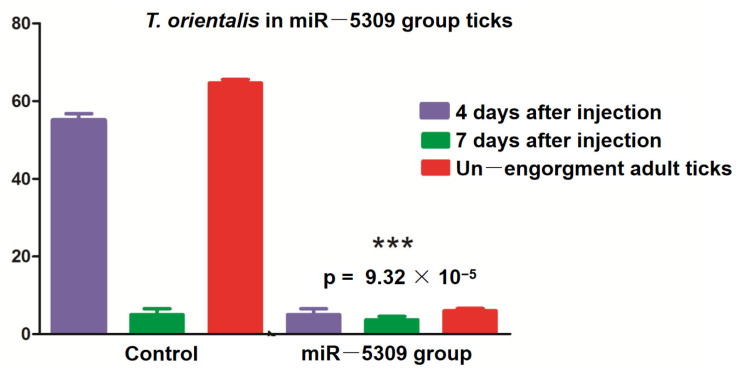
Ticks were microinjected with a miR-5309 antagomir, and samples obtained from nonengorged adult ticks were assessed on the fourth and seventh days by RT–PCR. The percentage of *T*. *orientalis* in the ticks in the miR-5309 group that had molted to become adults was significantly reduced (*p* = 9.32 × 10^−5^). *** indicates a significant difference between factors “4 days after injection” and “Un—engorgment adult ticks”.

**Figure 7:**
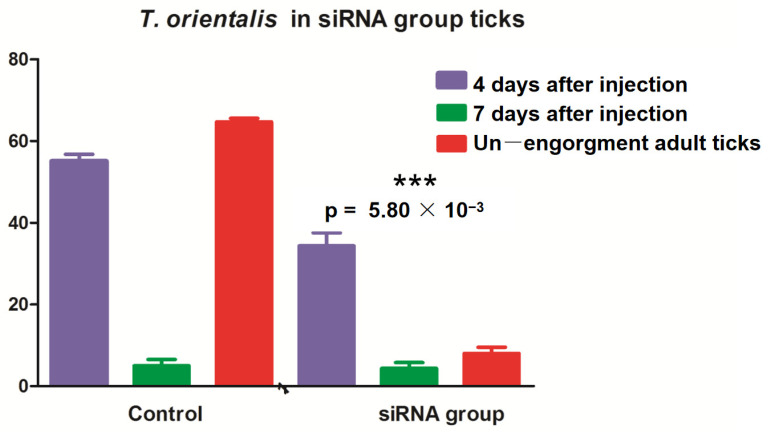
Ticks were microinjected with short interfering RNA (siRNA) targeting longipain, and the samples obtained from nonengorged adult ticks were assessed on the fourth and seventh days by RT–PCR. The expression of *T*. *orientalis* in the ticks in the siRNA group that had molted to become adults was significantly reduced (*p* = 0.0058). *** indicates a significant difference between factors “4 days after injection” and “Un—engorgment adult ticks”.

The larvae and nymphal ticks were infected with *T. orientalis* from the blood of cattle and collected after 1–8 days. These ticks were randomly selected for allocation into three groups, with 30 ticks per group. RNA was extracted, and RT–qPCR was performed for *T*. *orientalis* detection. The results revealed *T*. *orientalis* in nonengorged *H. longicornis* nymphal ticks on the fifth day, but Piroplasmida was detected as the adult ticks continued to suck blood. According to the test results, the number of *T*. *orientalis* larvae in the miR-5309-injected group that molted to become adult ticks was significantly lower than that in the control group (Figure 6). These results preliminarily confirmed that the low expression of miR-5309 promoted *Theileria* infection in these ticks. In addition, RT–qPCR was performed on samples obtained from infected nymphs and adult ticks after three injections of the short interfering RNA (siRNA) si-hlo-longipain. This result showed that miR-5309 expression was significantly lower in the adult ticks in the *T. orientalis*-infected siRNA-treated group than in those in the 4-day injection group (Figure 7), which preliminarily confirmed that the longipain gene plays a certain regulatory role in *T*. *orientalis*-infected *H*. *longicornis.*

## 4. Discussion

To date, the function of miRNAs during tick infection has not been adequately studied, and few reports exist in the literature. Therefore, our identification and analysis of the differential expression of miRNAs in *H. longicornis* infected with *T. orientalis* expand our understanding of miRNAs and elucidate the mechanisms and processes underlying tick transmission, providing new research ideas for effective transmission control. In this study, the expression levels of miRNAs were detected in infected ticks. To ensure the reliability of our data analysis, the sRNA distribution was determined based on length using clean reads (Figure 1). The results showed two significant peaks for the distribution of tick-derived sRNA lengths: One peak indicated a length of 22 nt, and the other peak indicated a length of 27–28 nt. This difference is a result of sRNA digestion by Drosha and Dicer enzymes during RNA processing. In addition, the length distribution provides insight into the distribution characteristics of different types of sRNAs, such as miRNAs ranging from 28 to 22 nt, siRNAs of 24 nt, and piwi-interacting RNAs (piRNAs) of 30 nt. These results show the quality and reliability of the data obtained by sequencing in this study, indicating their appropriateness for use in experimental analysis.

Differential expression is an important piece of information to consider when analyzing differences in the expression of factors in different treatment groups. In this study, miRNAs were predicted from sRNAs using rules established for miRNA identification [10], and the candidate miRNAs were compared between all species in the miRBase database (https://mirbase.org, accessed on 5 March 2023). After comparison and analysis, the miRNAs of tick origin were ultimately identified. Differential expression analysis of miRNAs was performed with DESeq2 software. Several miRNAs, including miR-5309, miR-2a, and miR-178, exhibited differential expression on the order of millions. In particular, the miR-5309 copy number was as high as 4.3 × 10^−175^ in *H. longicornis* infected with *T. orientalis*, and this value was markedly greater than that found for the uninfected group. These results indicate that miR-5309 may be involved in the process of infection and may play an important role in defense [36]. To verify this hypothesis, we used TargetScan (http://www.targetscan.org/vert_72/, accessed on 5 March 2023) to predict the possible target genes of miR-5309. These results suggested that longipain, an important target gene, is involved in the response of ticks to pathogen invasion. To confirm this hypothesis, GO enrichment analysis was performed (Table 4). The results showed that the cellular component of longipain consists mainly of a caspase complex; thus, longipain is likely involved in the regulation of cysteine-type endopeptidase activity (both positive and negative) [37]. Experiments were conducted to explore the regulatory role of miR-5309 during *T. orientalis* infection in *H. longicornis*.

The expression of the miR-5309 and longipain genes at different developmental stages in *H*. *longicornis* infected and not infected with *T*. *orientalis* was detected by RT–qPCR, and the results indicated that the expression abundance of miR-5309 in infected ticks was significantly greater than that in uninfected ticks. Its expression is greater in nonimmune organs (epidermis, Martensian tubules) in tissues, but its expression in immune organs (midgut, salivary gland) is significantly greater in other tissues (Figure 4). The expression of longipain increases with the development and maturation of the tick, and its expression in the midgut is significantly greater than that in other tissues. These findings demonstrated that the miR-5309 and longipain genes play a certain regulatory role in the process of infection. Notably, there is currently no information on the antigenic effect of miR-5309 in regulating the longipain gene. However, some studies have confirmed that miRNAs, such as miRNA-30e, can regulate the SOCS1 and SOCS3 genes during bacterial infection [38]. MiR-5309 may play a positive regulatory role in longipain gene expression. Moreover, the longipain expression levels in various *H*. *longicornis* tissues were measured, and the results showed that the levels were greater in the midgut, which is consistent with the findings reported by Tsuji N [29]. The midgut is an important digestive and immune organ in ticks that can secrete many immune factors and participate in the identification and elimination of pathogens [39]. On the one hand, these factors protect the host from pathogen invasion, and on the other hand, pathogen surface proteins bind to host cell surface factors to obtain the materials needed for survival [40]. However, the abovementioned studies only analyzed the functional issues of some genes and did not truly address the interactions between genes and pathogens. In the future, we will further explore the molecular mechanisms underlying the interactions between tick-derived genes and pathogens to provide a reference for the study of Theileria vaccines.

In this study, miRNA inhibition and RNA interference (RNAi) experiments were performed using *H*. *longicornis* infected with *T*. *orientalis*. The treated ticks were cultured from cattle infected with *T*. *orientalis*, and clean ticks from uninfected cattle were used as controls. During the process of engorging and molting into the subsequent developmental stage after engorging, these ticks were randomly selected based on extracted RNA reverse-transcribed to cDNA. The tick samples were assessed for the detection of *T*. *orientalis* infection. The expression of the longipain target gene in the siRNA and miR-5309 groups decreased significantly after the ticks molted and entered the adult stage, which preliminarily indicated that the longipain gene and miR-5309 play a certain role in the replication of *T*. *orientalis* in *H*. *longicornis*. However, further experiments are needed to determine the specific immune mechanism involved.

## 5. Conclusions

This study provides the first clarification that miR-5309 plays a positive regulatory role in *T*. *orientalis* infection in *H*. *longicornis*. The target gene longipain affects the infectivity of *T*. *orientalis*. These findings are expected to facilitate in-depth studies of the interaction mechanism between ticks and tickborne pathogens and to provide ideas for the development of measures for the prevention and control of tickborne diseases, including those involving the use of prevention and control agents.

## Figures and Tables

**Table 1:** List of primers used in this study.

Primers Name	Primer Sequence (5′–3′)
Stem–loop primer	GTCGTATCCAGTGCAGGGTCCGAGGTATTCGCACTGGATACGACTTCGGGG
β-*Actin* sense primer	GTTCCTGGGTATGGAATCG
β-*Actin* antisense primer	TCCACGTCGCACTTCATGAT
miR5309 F	ACACTCCAGCTGGCAATGCCCATGGAA
miR5309 R	GTCGTATCCAGTGCAGGGTCC
longipain-qPCR-F	CAACTCCTGGAACACCGAATGGG
longipain-qPCR-R	TCGTCTTCAATGCCGCACTCATC
si-hlo-longipain	GGCGTATTACAATCCGAAA

**Table 2:** Differential expression analysis between clean and infected ticks.

miRNA Name	Count (HLTor)	Count (HLNC)	TPM (HLTor)	TPM (HLNC)	log2 Ratio (NC/Tor)	Up- or Downregulation	*p* Value
miR-5309	130	1804	22.88	180.57	1.8161	Up	8.84 × 10^−193^
miR-2001	1324	5229	233.01	523.98	1.1691	Up	1.69 × 10^−176^
miR-96	99	1305	17.42	130.77	2.9082	Up	1.82 × 10^−145^
miR-184	67,803	138,893	11,932.56	13,917.91	0.2220	Up	1.17 × 10^−140^
miR-5312	63	1090	11.09	109.22	3.2999	Up	8.67 × 10^−137^
miR-96	906	5238	155.75	343.04	1.1391	Up	5.64 × 10^−127^
miR-5315	207	1329	36.43	133.17	1.8701	Up	1.72 × 10^−89^
miR-5310	218	1199	38.37	120.15	1.6467	Up	2.21 × 10^−68^
miR-993	1839	1429	240.88	97.05	−1.3115	Down	3.84 × 10^−147^
miR-2a	1339	1180	235.65	118.24	−0.9949	Down	1.58 × 10^−66^

Note: “HLTor” indicates that *Hemaphysalis longicornis* was infected with *Theileria orientalis*, “HLNC” is the control for clean *Hemaphysalis longicornis*, “HL” refers to *Hemaphysalis longicornis*, “Tor” is *Theileria orientalis*, and “NC” is the control. “TPM” (transcripts per million) refers to the number of transcripts per million.

**Table 3:** Putative miR-5309-binding sites in longipain.

miRNA	Site	Free Energy	Binding Site
miR-5309	1122	−27.8 kcal/mol	Target 5′ C U GAUGA C A 3′ UUCG GGG UCC GUG GGCAUUG AAGC CCC AGG UAC CCGUAAC miRNA 3′ AA 5′

**Table 4:** GO enrichment analysis of target genes of miR-5309.

Cellular Components	Molecular Functions	Biological Processes
caspase complex	cysteine-type endopeptidase inhibitor activity	negative regulation of cysteine-type endopeptidase activity
	cysteine-type endopeptidase activity involved in apoptotic process	positive regulation of cysteine-type endopeptidase activity
	GPI-anchor transamidase activity	regulation of cysteine-type endopeptidase activity
	cysteine-type endopeptidase activator activity	/
	calcium-dependent cysteine-type endopeptidase activity	/
	ubiquitin-like protein-specific endopeptidase activity	/

Note: “/” indicates that the target gene to the corresponding miRNA has not been annotated with biological processes.

## Data Availability

The data that support the findings of this study are available upon reasonable request from the authors.
