# Correction: Luo et al. Role of Recognition MicroRNAs in *Hemaphysalis longicornis* and *Theileria orientalis* Interactions. *Pathogens* 2024, *13*, 288

**DOI:** 10.3390/pathogens13060502

**Published:** 2024-06-13

**Authors:** Jin Luo, Yangchun Tan, Shuaiyang Zhao, Qiaoyun Ren, Guiquan Guan, Jianxun Luo, Hong Yin, Guangyuan Liu

**Affiliations:** 1 State Key Laboratory for Animal Disease Control and Prevention, Key Laboratory of Veterinary Parasitology of Gansu Province, Lanzhou Veterinary Research Institute, Chinese Academy of Agricultural Science, Xujiaping 1, Lanzhou 730046, China; lxt9093@163.com (Y.T.); zhaoshuaiyang@caas.cn (S.Z.); renqiaoyun@caas.cn (Q.R.); guanguiquang@caas.cn (G.G.); luojianxun@caas.cn (J.L.); 2 MOE Joint International Research Laboratory of Animal Health and Food Safety, College of Veterinary Medicine, Nanjing Agricultural University, Nanjing 210095, China; 3 Jiangsu Co-Innovation Center for the Prevention and Control of Important Animal Infectious Disease and Zoonosis, Yangzhou University, Yangzhou 225009, China

## Addition of Two Authors and One Affiliation

In the original publication [1], there was a mistake in the authorship. Yangchun Tan and Guangyuan Liu were not listed in the authorship; Guangyuan Liu should have been listed as one of the corresponding authors, and Yangchun Tan should have been listed as one of the first authors. The new affiliation “MOE Joint International Research Laboratory of Animal Health and Food Safety, College of Veterinary Medicine, Nanjing Agricultural University, Nanjing 210095, China” was added as affiliation 2. 

The updated Author Contributions statement is below:

**Author Contributions:** J.L. (Jin Luo), G.L., and S.Z. designed the experiments. Q.R., S.Z., G.G., and Y.T. performed the experiments. J.L. (Jin Luo) analyzed the data. J.L. (Jianxun Luo), H.Y., and J.L. (Jin Luo) critically revised the manuscript. J.L. (Jin Luo) wrote the manuscript. All authors have read and agreed to the published version of the manuscript.

The authors state that the scientific conclusions are unaffected. This correction was approved by the Academic Editor. The original publication has also been updated.
